# Application of artificial neural networks for automated analysis of cystoscopic images: a review of the current status and future prospects

**DOI:** 10.1007/s00345-019-03059-0

**Published:** 2020-01-10

**Authors:** Misgana Negassi, Rodrigo Suarez-Ibarrola, Simon Hein, Arkadiusz Miernik, Alexander Reiterer

**Affiliations:** 1 Department of Sustainable Systems Engineering INATECH, University of Freiburg, Emmy-Noether-Straße 2, Freiburg, Germany; 2 Department Object and Shape Detection, Fraunhofer Institute for Physical Measurement Techniques IPM, Heidenhofstraße 8, Freiburg, Germany; 3 Department of Urology, Faculty of Medicine, University of Freiburg-Medical Centre, Hugstetter Str. 55, Freiburg, Germany

**Keywords:** Neural networks, Deep learning, Cystoscopic images, Medical image analysis

## Abstract

**Background:**

Optimal detection and surveillance of bladder cancer (BCa) rely primarily on the cystoscopic visualization of bladder lesions. AI-assisted cystoscopy may improve image recognition and accelerate data acquisition.

**Objective:**

To provide a comprehensive review of machine learning (ML), deep learning (DL) and convolutional neural network (CNN) applications in cystoscopic image recognition.

**Evidence acquisition:**

A detailed search of original articles was performed using the PubMed-MEDLINE database to identify recent English literature relevant to ML, DL and CNN applications in cystoscopic image recognition.

**Evidence synthesis:**

In total, two articles and one conference abstract were identified addressing the application of AI methods in cystoscopic image recognition. These investigations showed accuracies exceeding 90% for tumor detection; however, future work is necessary to incorporate these methods into AI-aided cystoscopy and compared to other tumor visualization tools. Furthermore, we present results from the RaVeNNA-4pi consortium initiative which has extracted 4200 frames from 62 videos, analyzed them with the U-Net network and achieved an average dice score of 0.67. Improvements in its precision can be achieved by augmenting the video/frame database.

**Conclusion:**

AI-aided cystoscopy has the potential to outperform urologists at recognizing and classifying bladder lesions. To ensure their real-life implementation, however, these algorithms require external validation to generalize their results across other data sets.

## Introduction

Bladder cancer (BCa) accounts for approximately 7% of all newly diagnosed cancers in the USA, being the fourth most common cancer and eighth most lethal in men [1, 2]. In Germany, BCa represents 4.7% of all new cancer cases and 3.2% of all cancer-related deaths [3]. Despite a low stage and grade, recurrence rates range from 50 to 75% at 20 years, setting patients at risk of progression to muscle-invasive disease [4]. Periodic cystoscopic examinations remain the cornerstone of patient follow-up to detect early recurrences and reduce the risk of progression [5]. However, cystoscopic findings are diverse and often challenging to recognize and classify, ranging from healthy tissue to urothelial carcinoma [6]. A precise recognition of these features currently depends on the examiner´s skill and experience, leading to wide inter-observer variability in the interpretation of cystoscopic findings [7].

Artificial intelligence (AI) is becoming more prevalent in the field of medicine as a critical component of computer-aided diagnosis (CAD) and subsequent treatment. This has been notably shown in the successful application of deep learning (DL) approaches in various medical image analysis tasks [8]. Such works include image classification, segmentation, detection and registration of data obtained from radiology, pathology and endoscopy. The emergence of AI-assisted endoscopy, by training DL algorithms with large data sets of images or videos, has shown promising results, providing CAD tools that improve lesion detection and achieve diagnosis with high sensitivity and specificity [9, 10]. AI-assisted endoscopy offers the potential to radically change surgical practice by assisting physicians in identifying areas of malignancy given the heterogeneous aspect of lesions [11]. In the field of urology, cystoscopy, in particular, can benefit from this methodology. The applicability of CAD in cystoscopic examinations, however, has not been extensively evaluated.

This work provides a brief overview of new methods for tumor visualization, current techniques of automated image evaluation and finally discusses novel AI-based methods to automatically evaluate cystoscopic images.

## Evidence acquisition

A comprehensive review of current literature was performed using the PubMed–Medline database up to October 2019 using the term “cystoscopy”, combined with one of the following terms: “machine learning”, “deep learning” and “convolutional neural network”. To capture recent trends in machine learning (ML), deep learning (DL) and convolutional neural network (CNN) applications, the search was limited to articles and abstracts published within the last 5 years, originally published in English. Review articles and editorials were excluded. Publications relevant to the subject and their cited references were retrieved and appraised independently by two authors (M.N. and A.R.). After full text evaluation, data were independently extracted by the authors for further assessment of qualitative and quantitative evidence synthesis. The following information was extracted from each study: name of author, journal and year of publication, AI method, number of participants per study, and outcome prediction accuracy.

## State of the art of urological endoscopy

Although white light cystoscopy (WLC) is the current standard of care for the initial evaluation of BCa, it has several shortcomings, such as the difficulty to detect small or flat lesions, including carcinoma in situ (Cis) [12]. Current data suggest that early recurrence in BCa patients may be the result of undetected lesions during cystoscopy and transurethral resection of bladder tumors (TURB) [13]. The application of advanced optical techniques is becoming more widespread to improve the diagnostic sensitivity of inconspicuous lesions and prevent recurrent non-muscle invasive bladder cancer (NMIBC).

In photodynamic diagnosis (PDD) or fluorescence cystoscopy, an optical imaging agent is instilled preoperatively into the bladder resulting in the accumulation of protoporphyrins in rapidly proliferating cells such as malignant bladder tumors [14]. They are subsequently converted to photoactive porphyrins emitting a red fluorescent light under blue light. Studies have demonstrated that PDD improves Cis detection and reduces recurrences in patients with known or suspected NMIBC thereby also reducing health-care costs [15]. Its role in the surveillance scenario is emerging for use in patients with high-risk tumor lesions [16].

Narrow band imaging (NBI) has been similarly designed to improve BCa detection compared to WLC. This technology aims to augment the visualization of tumoral blood vessels by light modulation, by narrowing the bandwidth of light output to 415 and 540 nm, which is strongly absorbed by hemoglobin [17].

Confocal laser endomicroscopy (CLE) couples microscopic imaging with a fiber-optic bundle transmitting a 488 nm laser light, which provides real-time and in vivo histopathologic information [18]. The CLE probe has a 0.85 mm to 2.6 mm diameter, 240 μm penetration depth and can pass through the working channel of standard cystoscopes [19]. It uses fluorescein as a contrast agent and can obtain a thorough evaluation of tissue structures to distinguish between low- and high-grade BCa. Unfortunately, a small field of view and reduced tissue penetration limit its ability for surveying the entire bladder. Moreover, large-scale studies examining the diagnostic accuracy of CLE, including sensitivity and specificity, are lacking.

Furthermore, new techniques and methods are currently under development: optical coherence tomography (OCT) is a non-invasive real-time microscopic imaging technique using a near-infrared wavelength light (890–1300 nm) with a 2 mm penetration depth that produces an ultrasound-like image [20]. OCT measures changes of backscattered light with an interferometer caused by asymmetric structures within the tissue and can be applied to standard rigid cystoscopy given its 2.7 mm diameter probe [20]. Huang et al. recently performed a meta-analysis to evaluate the diagnostic accuracy of OCT. The sensitivity and specificity of the included publications ranged from 76 to 100% and 62% and 97%, resulting in a pooled sensitivity and specificity of 0.96 and 0.82, respectively [21]. OCT is limited by a small area of analysis, making the examination of the entire bladder impractical; therefore, its use with other ancillary methods is encouraged.

Raman spectroscopy (RS) is an endomicroscopic technology that does not require photoactive substances and can be used to examine molecular components in tissue. RS uses infrared light (785–845 nm) to analyze the inelastic photon scattering after its interaction with intramolecular bonds. RS can determine the integrity of bladder wall layers, assess penetration depth and recognize low- or high-grade BCa [19]. Chen et al. recently applied a fiber-optic Raman sensing probe to evaluate the diagnostic potential of RS in identifying various bladder pathologies ex vivo. The sensitivities and specificities for normal bladder tissue and low-grade and high-grade bladder tumors were 88.5%, 95.1% and 90.3%, and 98%, 97.5% and 96.4%, respectively [22]. Nevertheless, RS has several limitations, such as a restricted field of view, making the screening of the entire bladder surface impractical and should be targeted at suspicious lesions identified by PDD or NBI.

Multiphoton microscopy (MPM) uses a laser-scanning microscope and simultaneously absorbs two near-infrared photons (700–800 nm) based on the autofluorescence of cells and extracellular components with intrinsic tissue fluorophores, such as flavin adenine dinucleotide (FAD) and nicotinamide adenine dinucleotide (NADH) to provide information on cellular metabolic activity [23]. Pradère et al. recently assessed an optical multimodal technique on samples of patients suspected of having BCa and found that it was able to discriminate tumor from healthy tissue and determine the grade of tumors [24]. However, when MPM is used alone, it is limited by its shallow penetration and the difficulty to recognize intranuclear modifications.

As opposed to the previously mentioned optical techniques, which require a human professional for interpretation, AI methods rely on training CNNs with images or videos so it may learn to recognize complex patterns. Although research in this area has increased dramatically in recent years, there is presently no real-time application of AI-assisted cystoscopy in the clinical setting to accurately and reproducibly classify cystoscopic findings.

## Artificial neural networks for analysis of images: an overview

In image analysis, the term object recognition refers to the detection or classification of objects in image or video data. It describes whether or to what extent input data are similar to previously seen objects. Methods for object class recognition are usually based on a learning process. Classical image processing methods such as SIFT [25] determine from a training data set a suitable selection of features that can be used to reliably identify instances of the object class. In recent years, object recognition methods using deep learning (DL) or artificial neural networks (ANNs) have shown to be superior to classical methods [26]. ANNs have a strong representational power that enables them to learn complex hierarchical representation of images, thus increasingly representing abstract concepts. They are powerful at generalizing to never seen data. This is especially the case when it comes to recognizing a multitude of different objects whose appearance also varies greatly. ANNs are also more robust against variations, e.g., masking, fading of colors, damage or soiling. As early as the end of the 1990s, ANNs were used to solve simple tasks such as handwriting recognition (so-called LeNet) [27]. DL differs from the classical methods for object recognition primarily in that the objects or object classes to be recognized are not described by a set of manually predefined features, but the feature representation of an object is learned during training from the neural network.

### Artificial neural networks

**Fig. 1:**
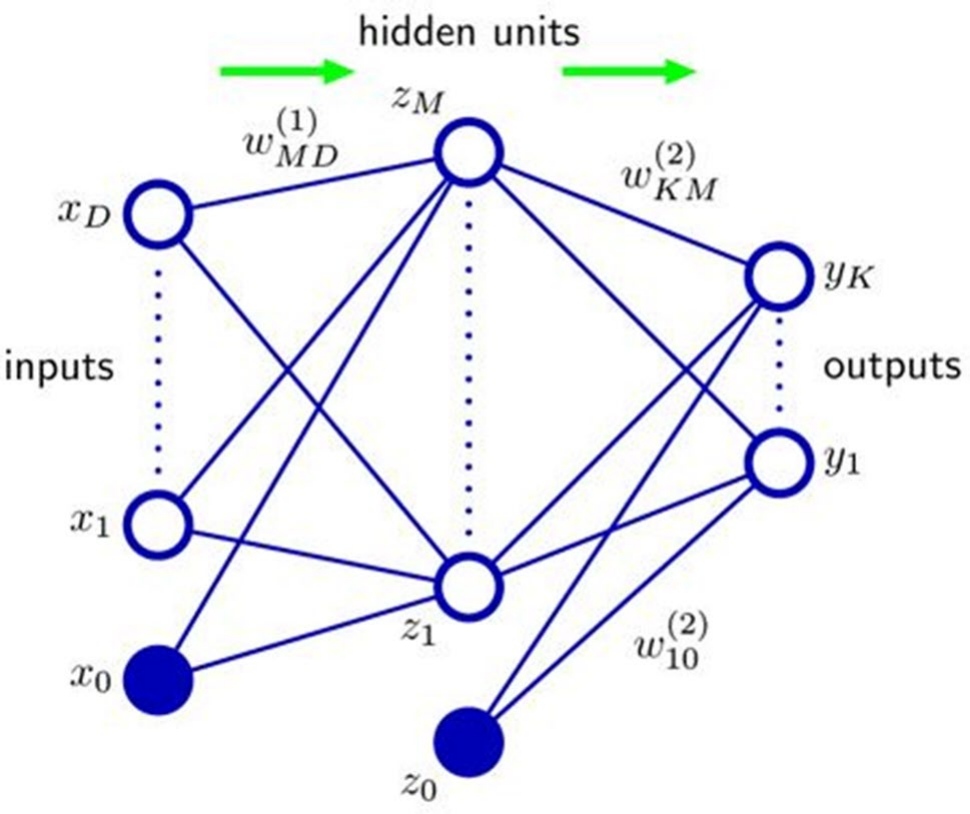
Multilayer perceptron with input and output layer as well as an additional hidden unit layer. The neurons of a layer are represented as unfilled blue circles, and the completely colored neurons are the so-called bias neurons, whose value is constant and does not depend on the activations in previous layers. The weights of the compounds are learned in training (Source: [1])

Most current DL approaches are based on a network architecture known as feedforward neural network or multilayer perceptron (MLP) [28, 29]. These networks use the backpropagation method for the actual learning process, which is also referred to as training. Figure 1 shows the architecture of a simple MLP. Each layer of the neural network consists of a set of neurons. The number of input neurons is chosen according to the input data, and the number of output neurons according to the target result; e.g., the input corresponds to the number of pixels in the image, and the output to the number of object classes to be detected. Between the input and the output layer, there are any number of hidden layers, called hidden units in literature. The layers are arranged vertically in Fig. 1; the green arrows symbolize the direction in which the information is passed through the network in a forward propagation. Each neuron is connected to all neurons from the previous layer. The activation of a neuron is thus determined from all activation values in the preceding layer. These are multiplied by the weights of the individual connections to this neuron. The weights are the parameters learned during the training.

The selection of the activation function depends on the application and represents one of the hyperparameters that influence the performance of the neural network. Typical activation functions are, e.g., Rectified Linear Unit (ReLU), Logistic Sigmoid Activation Function or Hyperbolic Tangent (tanh). The activation function must be differentiable so that the derivation or gradient can be determined during training. The activations in subsequent hidden layers are calculated analogously. In summary, the output values are calculated from the input values for the MLP.

Although the MLP seems simple in its structure, many tasks can be solved with it. Thus, any limited continuous function can be approximated with any accuracy by an MLP with only one hidden layer [30]. Based on this simple network structure, a variety of network architectures for different applications have been developed.

### Convolutional neural networks

In the field of image processing, the so-called convolutional neural networks (CNNs) are of particular interest [31]. These networks take advantage of the fact that information in images is mostly local, i.e., limited to a smaller part of the image. They exploit this spatial layout of images by using local connectivity and parameter sharing [32]. Therefore, filters can be used for the analysis of the image content, which only look at a small part of the image. This means that a much smaller number of neurons is required than is the case for fully connected networks, which results in considerable reduction in computing time and memory requirements. However, it is only in recent years that it has become possible to develop networks that can handle huge amount of input data for the detection of more complex objects. This is due in particular to the availability of powerful and highly parallel graphics processors (graphical processing units, GPUs) [26]. CNNs have played a major role in the success of DL for image analysis in a wide range of applications including image classification [26, 33, 34], image segmentation [34], instance segmentation [34] and object detection [35, 36].

**Fig. 2:**
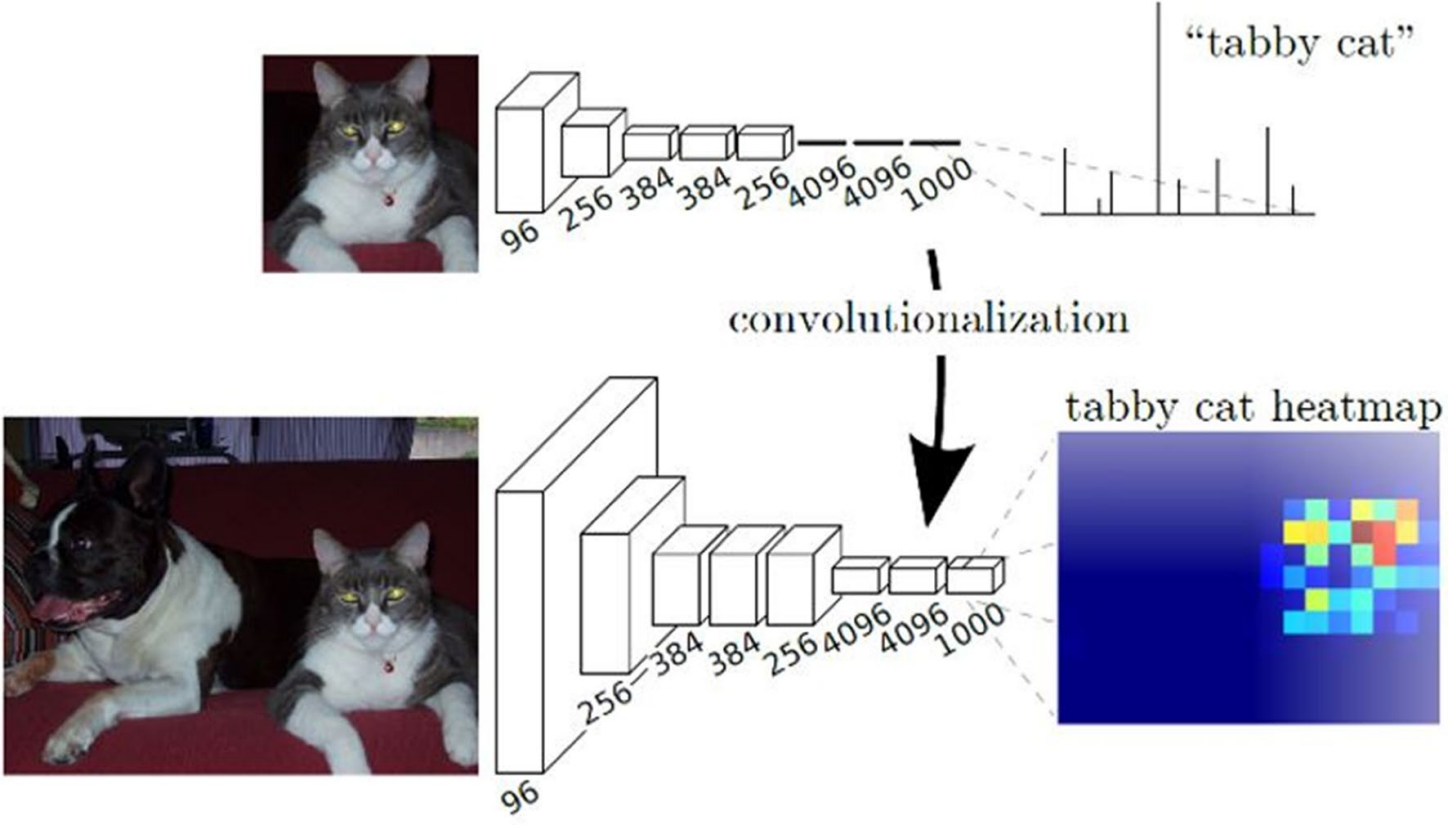
Schematic comparison of the architecture of two neural networks. Above: network for classification; below: network for calculating the approximate localization of an object using the activations of the feature map (heat map). The numbers below the layers represent the number of feature maps (Source: [29])

*Image classification* The breakthrough for such deep networks was achieved in 2012 with AlexNet [26]. This network achieved much better results for the classification of images from a benchmark data set than all classical methods for object recognition. The schematic structure of a network for classification is shown in the upper part of Fig. 2. Clearly visible is the reduction of the resolution with increasing depth of the network, with simultaneous increase of the feature space. By reducing the resolution at the transition between the layers of the network, an ever larger area of the image is also considered. In the end, there is usually an analysis of the entire image by several completely connected layers. The weights of the neurons are calculated during the training of the network using manually annotated training data. The weights are optimized by backpropagation, a special variant of the gradient descent method. At the end, the weights contain all the information necessary for the classification of objects. They decide how strongly a characteristic represented by a filter is weighted and how much neurons in subsequent layers are activated.

**Fig. 3:**
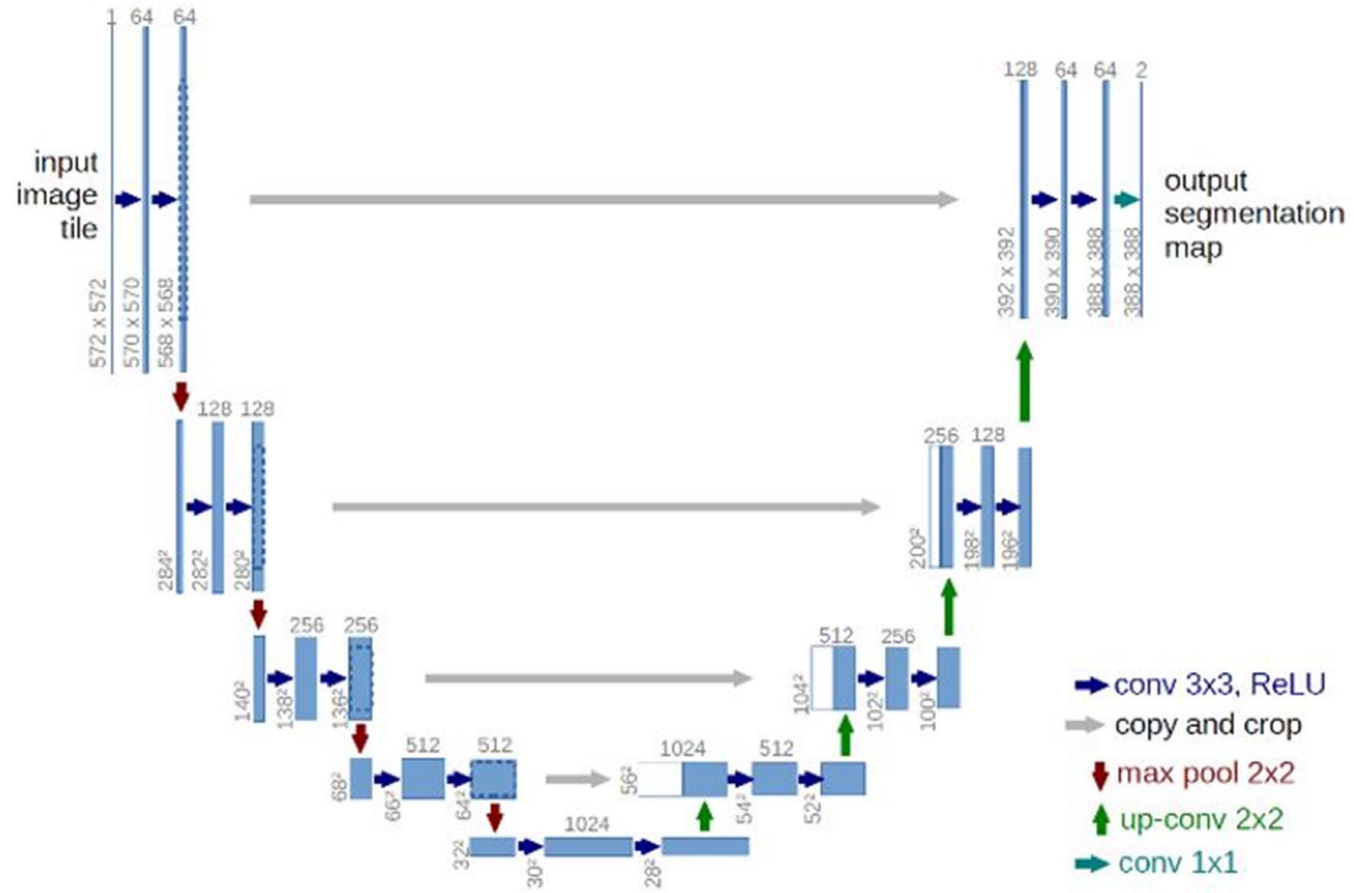
Schematic view of the architecture of an up-convolutional network for the semantic segmentation of images. The left part takes over the classification, while the information about the localization of objects is lost. The right part restores this information by upsampling (Source: [9])

*Image segmentation* CNNs are not only suitable for classifying an entire image (one class per image), but also for the challenging task of semantic segmentation. Such networks have a much more complex task, namely the classification of each pixel in an image, as compared to networks for image classification, where the output is to determine the probability of image belonging to a certain class. An architecture that has been proposed for this task is the so-called fully convolutional networks (FCN) [37]. These networks add further layers to the classification part, which upsample the feature maps to achieve segmentation maps that have the same resolution as the input data. An example of such an encoder–decoder network architecture is the U-Net [8], which is successfully used for segmenting biomedical images. U-Net is a simple but powerful network, it is fast to train and delivers precise segmentation [8, 38]. It requires fewer amount of data to achieve good results, while retaining the possibility to train on large sample sizes [39]. Since its introduction, it has achieved top ranks in various medical imaging tasks and its architecture is used as a basis in many DL models [38-44]. The encoder part of the U-Net network applies convolution and max-pooling operations to extract useful semantic information from images. The decoder part upsamples this information with the help of spatial information acquired using skip connections from higher-resolution feature maps in the encoder part [42]. The result is a precise segmentation, in other words per pixel classification, of structures in the given image. Figure 3 shows a schematic overview of this network architecture.

*Instance segmentation* A segmentation approach that combines object detection [35] and semantic segmentation [37], the so-called instance segmentation, is a rapidly growing application area of CNNs. The idea is to localize objects in an image and precisely segment each instance [45]. For medical image analysis, instance segmentation models such as Mask-RCNN are reported to be powerful at detecting instances accurately, but generate less accurate segmentation masks compared to U-Net [46].

An essential requirement for training neural networks is to have a sufficient amount of annotated training data. Manual annotation is time-consuming; for instance, in image segmentation each object must be traced exactly. Ideally, the training data and the test data should come from the same distribution. While it is possible to classify objects in similar scenes with networks pre-trained on data from related domains, a good quality training data is very crucial. Training on large amount of data is very computation intensive and can take several days to weeks even on powerful graphics cards [47, 48]. During inference, however, the fully trained networks usually carry out the analysis of an image quickly. Typical values are processing times from 5 ms per image for simple tasks up to approximately 500 ms for more complex analyses using a current GPU; of course, this depends on the degree of optimization of the algorithms, training procedures, and the performance of the hardware used [35, 47].

Another challenge in training DL models is their sensitivity to many hyperparameters that have to be set properly to achieve optimal results. Tuning these hyperparameters manually requires domain knowledge of their effect on the generalization of the network [32] and is time intensive. Significant progress has been done in automatic tuning of hyperparameters for DL models; however, the process is still computationally intensive [49-52].

**Fig. 4:**
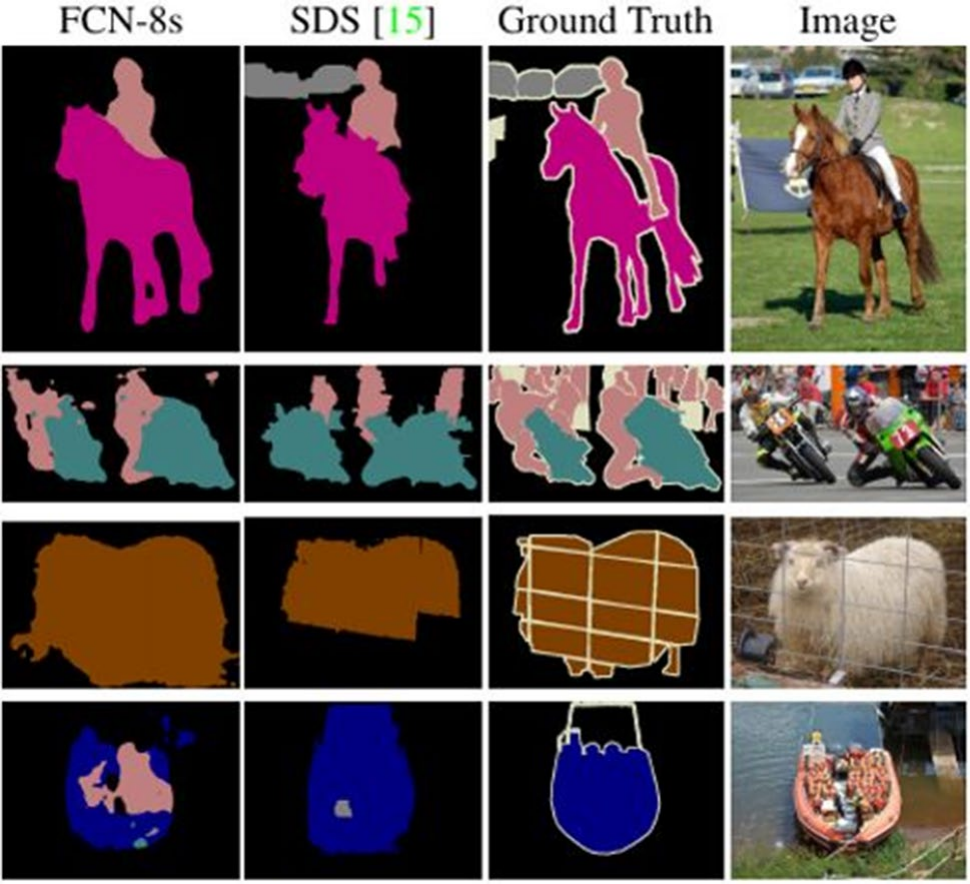
Semantic segmentation divides an image into semantically related regions by assigning each pixel of an image to a specific class (coded here by color). Ground truth is the manual annotation. The segmentation of complex scenes is still a challenge (Source: [31])

A clear advantage of neural networks is the possibility of end-to-end training and the fact that neural networks are very powerful at generalizing to never seen data. Exemplary results of two current approaches to semantic segmentation can be seen in Fig. 4.

## Application of ANNs for urological endoscopy

The field of urology has benefitted from recent advances in DL. However, in early stages, significant work has been done toward application of DL in areas such as image classification [26], object detection [35] and image segmentation. Ikeda et al. trained a CNN with 177 images of histologically confirmed bladder tumors and 133 images of healthy urothelium [53]. The neural network was pre-trained with 1.2 million images from ImageNet data set [54], which were classified into 1000 categories. The output revealed 93.0% sensitivity and 83.7% specificity for differentiating between tumoral and healthy urothelium.

Eminaga et al. studied 479 cystoscopy videos from patients with 44 urologic findings [6]. The authors used data augmentation to enlarge their data set of 479 images by up to a factor of 40, resulting in 18,681 images. The authors evaluated several CNN models and found that the Xception-based model achieved the highest accuracy score (99.52%) for the diagnostic classification of cystoscopic images. Nonetheless, 7.86% of bladder stone images and 1.43% containing indwelling catheters were falsely classified [6].

In a recent work, on automated detection of BCa on cystoscopic images, Shkolyar et al. used deep CNNs to detect suspicious lesions from cystoscopic videos [1]. The study uses TUMNet, an image analysis software based on DL to detect bladder papillary tumors on cystoscopic videos of 100 patients. The software first extracts video frames containing tumors and segments tumors within the selected frames. The authors report 90% sensitivity for tumor detection. Table 1 summarizes the current AI approaches in cystoscopic image analysis.

**Table 1:** Summary of current approaches of AI in cystoscopic images

Author	Training procedure	Data set	Prediction accuracy
Artificial neural networks on analysis of cystoscopic images			
Eminaga et al. 2018 [6]	Image classification, 44 classes	18, 681 training images, used data augmentation	Xception model: F1 score (99.52%). Other models 0.04–4.05 difference in performance to Xception model
Shkolyar et al. 2015 [1]	Instance segmentation, 2 classes: cancer and benign	Training with 417 cancer and 2335 normal frames, validation 211 cancer, 1002 normal frames	per-frame sensitivity was 88%, per-frame specificity was 99%, per-tumor sensitivity was 90%
Ikeda et al. 2018 [53]	Image classification, binary classes: tumoral and healthy urothelium	Data from 177 tumor lesion images and 133 normal images	93.0% sensitivity and 83.7% specificity

The impact of AI in interpreting cystoscopic images is currently under study in a recent collaborative research project, RaVeNNA-4pi. The goal of the project is to develop a digital platform with 4PI Endoimaging for 3D-reconstruction, semantic segmentation and visualization of the bladder for BCa documentation and improved patient follow-up.

**Fig. 5:**
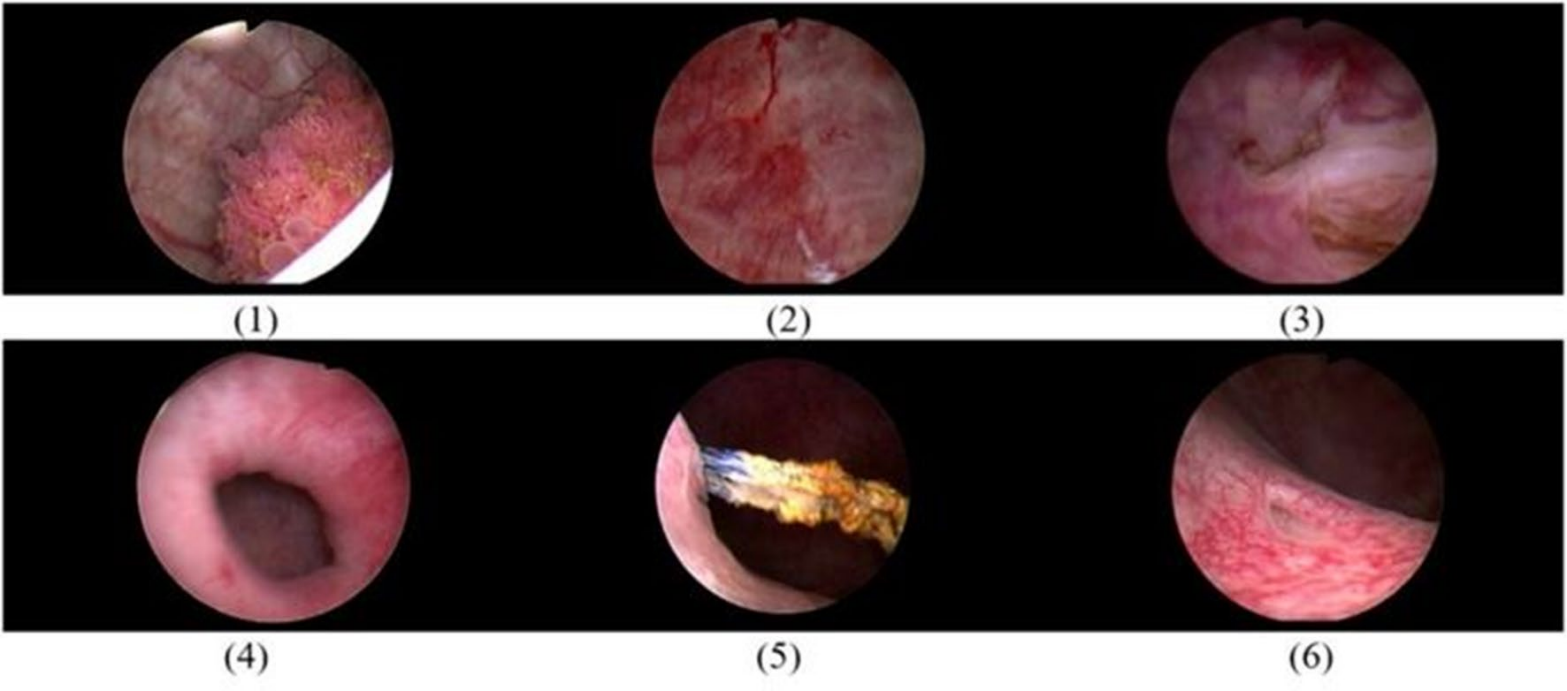
Representative images from RaVeNNA cystoscopy data set. From top left to down right, we show the classes: (1) papillary tumor, (2) ca in situ suspicion, (3) resented tumor, (4) diverticulum, (5) bladder stone, and (6) left and right ureteric orifice

RaVeNNA-4pi focuses on multi-class segmentation of cystoscopic images using DL. Semantic segmentation allows precise segmentation of classes of interest such as tumors in an image and is helpful in documentation and visualization of tumors in a 3D-reconstructed bladder. As part of the project, a novel data set of cystoscopic images was created and annotated by trained investigators. For the data acquisition process, patients´ cystoscopy videos were collected from cystourethroscopy procedures. These procedures were performed with a rigid cystoscope and a 30° lens under white light. Video frames of interest were extracted and manually annotated using a special label editor software (developed at the Fraunhofer Institute for Physical Measurement Techniques IPM). The annotation was conducted by two physicians and subsequently confirmed by a board-certified urologist. Classes of interest in this project are: (1) papillary tumor, (2) carcinoma in situ suspicion, (3) resected tumor, (4) bladder diverticulum, (5) bladder stone, and (6) left and right ureteric orifices. The RaVeNNA cystoscopy data set currently consists of 4200 annotated images extracted from 62 videos. An example of images is shown in Fig. 5.

The procedure uses U-Net, an encoder–decoder network that has been used successfully in various medical image analysis tasks [8, 38, 42]. The network architecture was slightly modified by adding batch normalization after each convolutional layer. To tackle class imbalance in the data, we use a weighted categorical cross-entropy loss. Data augmentation methods such as image rotation and zoom were used to make the network more robust.

**Fig. 6:**
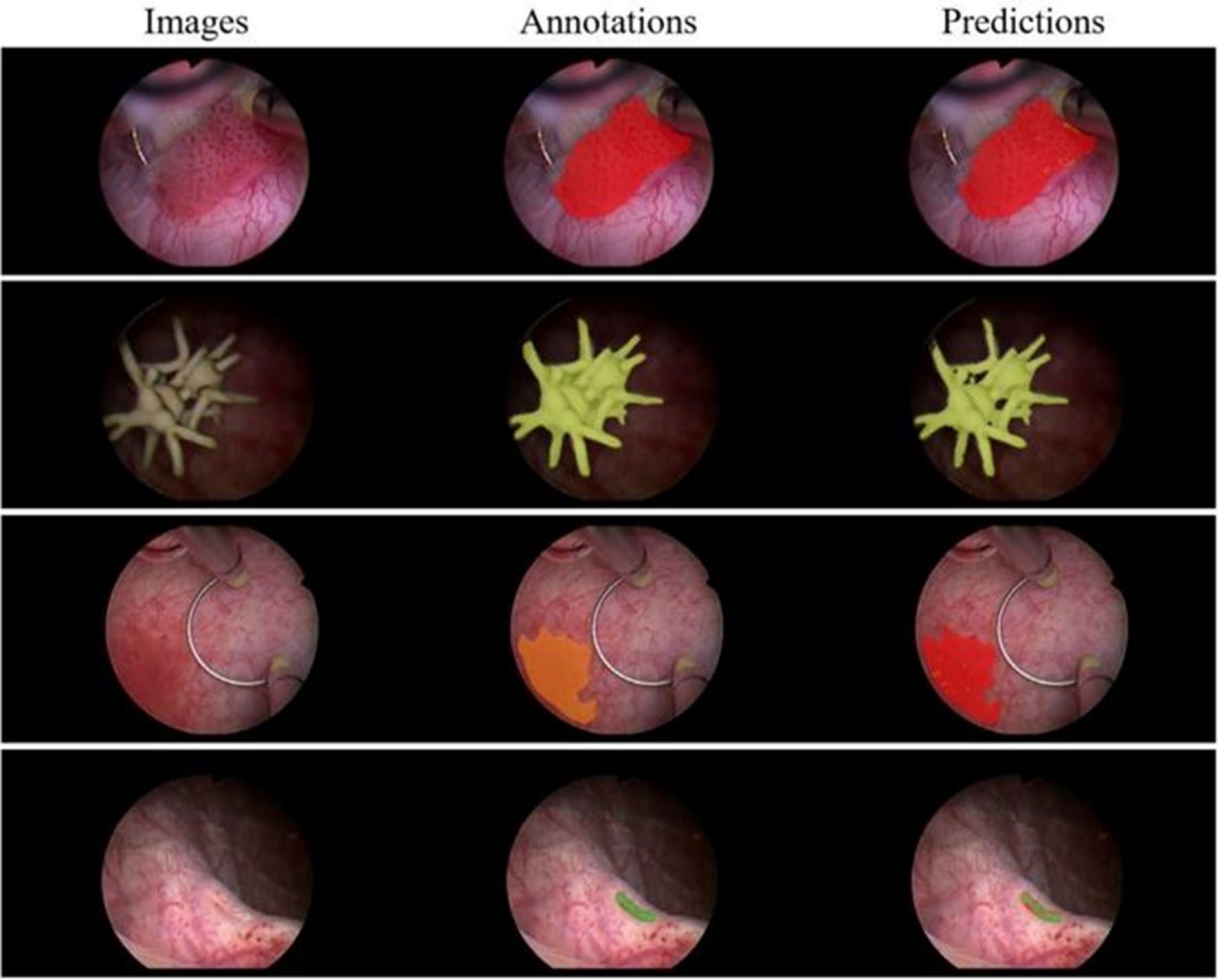
Representative images show results of the network. Each row shows an image from a specific class—papillary tumor, bladder stone, ca in situ and orifice—from top to bottom, respectively. On the far left are images, in the middle the manual annotations and on the right predictions of the network. The image in the third row is an example of how a papillary tumor and a bladder lesion suspicious for ca in situ can be misclassified

For training purposes, 70% of the data were used for training, 10% for validation, and 20% for test. The network achieved 0.67 average dice score coefficient (DSC). Examples of original images, annotation and automated segmentations are shown in Fig. 6. The study shows that deep CNNs can be used for semantic segmentation of multiple cystoscopic image categories.

We found that certain classes, such as papillary tumors and Cis suspicion, have high inter-class similarities and therefore are challenging for pixel-wise classification, and thereby degrade the average DSC performance over all classes. The current research work focuses on developing methods to distinguish these classes with higher precision/recall.

## Conclusion

Medical care is one of the most important areas of a modern society. The contributions made in this field in clinics, research and teaching have continuously improved the standard of living of citizens and increased life expectancy worldwide. In the recent past, medical research has been strongly influenced by the inclusion of IT methods and different microsystem technologies among diagnostic and therapeutic means. Machine learning (ML) enables highly efficient investigation of large data sets in which regularities and patterns are searched for. Particularly in cystoscopy, image-based data are generated that have not yet been adequately researched and exploited. In this work, outlines and potential of these technologies for application in urology were discussed. The next years will certainly be shaped by the incorporation of AI methods into result interpretation and reporting by endoscopic examinations. Scientific activities in this field should therefore be intensified.
